# Acoustic Sensor Network for Relative Positioning of Nodes

**DOI:** 10.3390/s91108490

**Published:** 2009-10-27

**Authors:** Carlos De Marziani, Jesus Ureña, Álvaro Hernandez, Manuel Mazo, Juan Jesús García, Ana Jimenez, María del Carmen Pérez Rubio, Fernando Álvarez, José Manuel Villadangos

**Affiliations:** 1. Electronics Department, University of Alcalá, Campus Universitario s/n, 28805, Alcalá de Henares, Madrid, Spain; E-Mails: alvaro@depeca.uah.es (A.H.);jesus@depeca.uah.es (J.J.G.);ajimenez@depeca.uah.es (A.J.M.);carmen@depeca.uah.es (M.C.P.R.);villa@depeca.uah.es (J.M.V.); 2. Electronics Department, National University of Patagonia San Juan Bosco, Facultad de Ingeniería, Ciudad Universitaria, Ruta Prov. N° 1, Km. 4, 9005 Comodoro Rivadavia, Chubut, Argentina; 3. Electrical Eng., Electronics and Automatics Department, University of Extremadura, Extremadura, 06071 Badajoz, Spain; E-Mail: fernando@depeca.uah.es

**Keywords:** sensor networks, relative localization, remote sensing

## Abstract

In this work, an acoustic sensor network for a relative localization system is analyzed by reporting the accuracy achieved in the position estimation. The proposed system has been designed for those applications where objects are not restricted to a particular environment and thus one cannot depend on any external infrastructure to compute their positions. The objects are capable of computing spatial relations among themselves using only acoustic emissions as a ranging mechanism. The object positions are computed by a multidimensional scaling (MDS) technique and, afterwards, a least-square algorithm, based on the Levenberg-Marquardt algorithm (LMA), is applied to refine results. Regarding the position estimation, all the parameters involved in the computation of the temporary relations with the proposed ranging mechanism have been considered. The obtained results show that a fine-grained localization can be achieved considering a Gaussian distribution error in the proposed ranging mechanism. Furthermore, since acoustic sensors require a line-of-sight to properly work, the system has been tested by modeling the lost of this line-of-sight as a non-Gaussian error. A suitable position estimation has been achieved even if it is considered a bias of up to 25 of the line-of-sight measurements among a set of nodes.

## 1. Introduction

The computation of relative positions among mobile computing devices [1] or members of a robot team [2] can provide enhanced support for multi-user interactions, when systems are not restricted to a particular environment and they cannot depend on any external infrastructure to compute their location, as is common in Local Positioning Systems (LPS) [3]. In these cases, it is necessary to design a sensor network capable of obtaining the distances among sensors in the shortest time and in a cooperative way. These kinds of systems are usually called Relative Localization Systems [4,5]; and the estimation of their coordinates is more difficult than in LPS, since positions are computed by measurements among objects whose locations are not known a priori in the environment.

Several positioning algorithms can be used to determine the relative position of objects, depending on different parameters: the accuracy required for the estimation, the number of observations available at each node and the computational load. In indoor spaces, multilateration techniques are often developed by solving a set of equations that consider all the measurements carried out among nodes. One of the most common algorithms in relative positioning systems is the classic or metric Multidimensional Scaling (MDS) technique [6]. This algorithm allows a geometric configuration of the considered objects with the smallest number of dimensions, by using only the distances measured among them. Nevertheless, data collected by the ranging mechanism are often influenced or corrupted by different parameters (environmental noise, signal delays associated to transducer responses, latency of signal processing algorithms, etc), which lead to errors in the estimation of coordinates. In order to reduce the error in the position estimation with the MDS algorithm, least-square minimization methods can be applied [7-9] using the geometric configuration provided by the MDS technique as starting point, and considering the different parameters associated to the ranging mechanism.

Many relative localization systems use data collected from a ranging mechanism, based on acoustic sensors, by measuring the times-of-flight (TOF) or difference-times-of-flight (DTOF) [8,9] in order to compute the coordinates. This often implies some additional links, e.g., infrared (IR) or radio frequency (RF), or accurate clock signals to measure the propagation time of the emitted signals, but increasing the complexity of the sensor nodes. In order to reduce the complexity of the sensor network, a low-complex ranging method, called Simultaneous Round-Trip-Time-of-Flight (S-RTOF), has been proposed by the authors [10]. This makes possible to determine temporary relations among the emissions carried out from every node using only acoustic signals. Additionally, because of the features of the ranging mechanism, a set of redundant observation data is obtained. This information can be useful to mitigate the non-line-of-sight effect (NLOS) that can appear when acoustic signals are used in indoor spaces. In this way, using high-level algorithms, such as geometry consistency check or statistical analysis [11,12], the estimator performance can be improved by identifying and rejecting those measurements with low accuracy.

In this work, the accuracy achieved in the position estimation by the proposed acoustic sensor network is analyzed. Object positions are computed by MDS algorithm, starting from the distance measurements. Finally, after considering the different possible errors and time delays involved in the computation of temporary relations, refined positions are computed by applying the Levenberg-Marquardt algorithm (LMA) to the non-linear equations which describe the temporary relations among emissions provided by the S-RTOF.

The rest of the paper is organized as follows: Section 2 shows the proposed acoustic sensor network architecture. Also, in Section 2, the used encoding scheme and S-RTOF mechanism are described, considering the different parameters involved in the measurement process. Section 3 explains the used positioning algorithms (MDS and LMA). In Section 4 Monte-Carlo simulations are done to determine the accuracy that can be obtained in the position estimation. Finally, some conclusions are discussed in Section 5.

## 2. System Architecture

**Figure 1.**
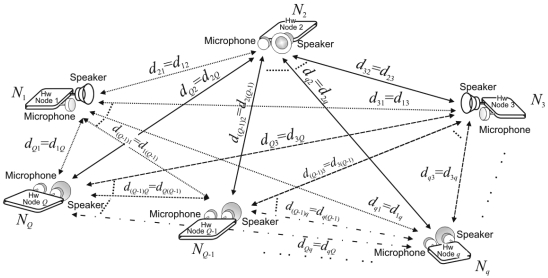
General scheme of the proposed sensor network.

The system architecture is depicted in Figure 1. It consists of an acoustic sensor network, where every object *N_q_* (*q* ∈ {1, 2,⋯,*Q*} and *Q* is the number of nodes in the system) carries a sensor node with a speaker, a microphone and the associated hardware. Acoustic sensing technologies are used since they are suitable for fine-grained location and widely spread due to their low cost, easy implementation and availability in assorted mobile systems.

The computation of the distances *d_ql_* (*q,l* ∈ {1,2,⋯,*Q*},*q* ≠*l*) between objects is carried out by measuring the propagation time of the acoustic signals emitted by every node, without any additional link to synchronize them. In order to simultaneously detect these acoustic emissions, a Code Division Multiple Access (CDMA) scheme has been used [13] by encoding the emissions with Complementary Sets of Sequences (CSS). A ranging mechanism has been developed based on the simultaneous Round-Trip-Time-of-Flight (RTOF) measurements from acoustic signals emitted by all the nodes. Since data are collected by all the nodes in a cooperative way, it is necessary to share the information obtained by every node in order to generate a map with all the object positions. In this case, data are distributed by means of low-cost RF communication modules [14], which are not synchronized with the acoustic ranging mechanism. It is not necessary any special requirement to collect and distribute the system information, so the proposed architecture can be implemented in general-purpose sensor or mobile computing systems. According to these features, every node can locally compute the object positions, providing a non-centralized architecture.

### 2.1. Encoding Scheme to Multi-User Detection

A remarkable capability for relative positioning is the measurement in a short time of all the spatial relations among objects, by using a common temporary reference. In order to measure the spatial relations among objects in the shortest time, it is necessary to use multi-user schemes that allow to simultaneously discriminate the emissions from every user or sensor.

In most cases, Direct-Sequence Code-Division Multiple-Access (DS-CDMA) techniques are used to discriminate the node emissions, by encoding every emitter with binary sequences and transmitting it by a simple phase modulation. These encoded signals are detected in a receptor by performing the correlation with every available sequence in the proposed system. Thus, the effectiveness depends on the properties of the used codes [13], requiring ideal properties of auto-correlation (AC) with high main-lobes for null time shifts, and side-lobes in the AC function closer to zero for all non-zero time shifts. Also, it requires that the values of the cross-correlation (CC) function among the different codes be as small as possible.

In the proposed system, an encoding scheme based on Complementary Set of *M* Sequences (*M*-CSS) has been used, where the number of sequences *M* is a power of two [15]. The features of this encoding technique allow to obtain *M* sets with ideal null cross-correlation, when the addition of the cross-correlation functions (ΣCC) between the corresponding sequences of two different sets is computed. Furthermore, the addition of the auto-correlation functions (ΣAC) from every sequence that composes the set provides null side-lobes. The described properties make attractive their use in sensory systems with simultaneous detection, since several emissions can be discriminated from different independent sources with low signal-to-noise ratios (SNR).

As opposed to the common encoding schemes used in localization, the encoding of emissions by *M*-CSS assigns more than one sequence to each user. In this way, the required hardware resources are increased, reason why efficient algorithms have been developed in order to minimize these requirements [16]. Furthermore, it is necessary to analyze the most efficient and effective technique to transmit in a short time the sequence of bits used to encode every user. One simple method consists of establishing an emission order of the *M*-CSS bits and transmitting them by a Binary Phase Shift Keying modulation (*BPSK*). An exhaustive analysis of this method has been made in [17], as well as some methods to reduce the worsening in the correlation properties provided by the proposed emission mechanism for simultaneous detection in a certain receptor.

**Figure 2.**
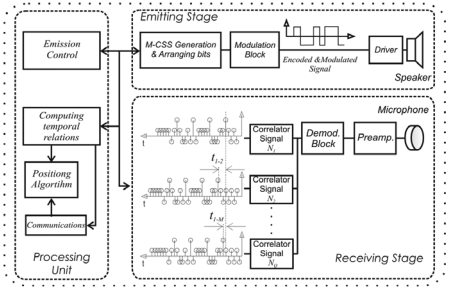
Detailed block diagram of node hardware architecture.

According to these signal processing techniques, the emitting stage implemented at the node hardware architecture is shown in Figure 2. This block is divided into the code generation stage for the *M*-CSS and the arranging bit stage. After that, the obtained encoding is transmitted by *BPSK*, which allows the emission to be adapted to the transducer bandwidth. Furthermore, a control emission block is implemented to fire the acoustic signal emission, managed by the processing unit. On the receiving module, the demodulation of the signal captured by the microphone is computed after adjusting and digitalizing it. Then, the signal is processed to determine the arrival time of every encoded acoustic emission. For that, a set of correlators has been implemented, according to the algorithms developed in [16] and [17], to detect the emissions carried out by, at most,*Q* nodes. With the results obtained at the correlator outputs, a peak detector algorithm has been applied in order to determine the arrival instant. Although *Q* nodes can be simultaneously detected in each common perception area, the *Q* codes can be replicated in nodes located in other different areas. Practical values of *Q* can reach, for example, 64 nodes depending on the time required between successive position computations.

Finally, a processing unit has been implemented at each node, which controls the emitting stage according to the results obtained at the correlator outputs. It also computes the temporary relations among the emissions carried out by every node. Moreover, this unit performs the distribution of the data collected by every node by means of a communication block, and it computes the position among the objects.

### 2.2. Principle of Measurements

Since all nodes are equal in their architecture and functionality, anyone of them can start the ranging process (it is called *Master* node). In this work, the *Master* node has been previously assigned (i.e., node *q* = 1) for a concise explanation of the measurement algorithm. Nevertheless, a multiple access technique as Carrier Sense Multiple Access (CSMA) can be used to automatically assign the master node.

**Figure 3.**
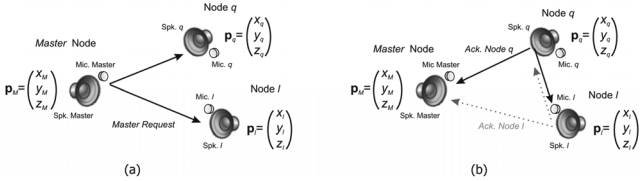
Principle of measurement using simultaneous Round-Trip-Time-of-Flight. (a) Emission of the request from the *Master* (starting the positioning process). (b) Acknowledgement from every node in order to compute distances.

It is assumed that node *q* = 1 is the *Master* and has the following coordinates (*x_M_, y_M_, z_M_*), described by a vector **p***_M_*. On the other hand, the positions of two slave nodes *q* and *l* are given by vectors **p***_q_* and **p***_l_*. At a given instant, the *Master* node emits its encoded acoustic signal (see Figure 3a) starting the RTOF measurement. This emission, called *Master Request*, is detected by every slave node with their microphones *Mic. q* and *Mic. l* at different instants according to their distribution in the environment.

In response to the *Master Request*, every slave node emits its own code, denoted as *Ack. Node*, which travels towards the *Master* node and also to the other slave nodes (see Figure 3b). In this way, in the *Master* node, the temporary interval from the *Master Request* emission until the detection of every *Ack. Node* can be computed. Also, taking advantage of the encoded emissions, it is possible to compute temporary relations in each slave node, from its own *Master Request* detection until the detection of the *Ack. Node* emissions from other slaves.

### 2.3. Pseudo-Time-of-Flight Equations

According to the described principle of measurement, it is possible to determine temporary relations among the emissions carried out from the different nodes, by measuring the differences between the correlation maximum values detected at every node. These values do not exactly describe the propagation time of the acoustic emissions, since there is an offset caused by the rising time of the emitted signal, the latency of signal processing algorithms, and the response time of the transducers [9]. Therefore, it is necessary to consider a delay until the signal is encoded and emitted by the speaker, denoted as *t_Spk_*. Also, in the reception stage, a delay *t_Mic_* appears from the capture of the signal by the microphone until obtaining the correlation maximum values. Assuming that the moving velocities of nodes are much smaller than the propagation speed of acoustic waves, it is possible to obtain (1), which summarizes the temporary relations measured in the *Master* node for every slave node:

(1) $${\hat{t}}_{M-q}=2\cdot \frac{\left\|{\text{p}}_{M}-{\text{p}}_{q}\right\|}{c}+tp_{M}+tp_{q}+T_{\text{CODE}}$$

where *t̂_M_*_−_*_q_* is the time computed between the *Master* (*q* = 1) and the slave node *q* with *q* ∈ {2,⋯,*Q*};*c* represents the propagation speed of sounds;*T*_CODE_ is the temporary length of the emitted encoded signal; ‖ ‖ is the Euclidean norm between the considered position vectors (**p***_M_* for the *Master* and **p***_q_* for the slave); and *tp* =*t_Spk_* +*t_Mic_* represents the aforementioned time delays (*tp_M_* and *tp_q_* for the *Master* and slave node *q* respectively). According to these features, the measurements have been called pseudo-times-of-flight (pTOF) [18], since they are not a direct time-of-flight measurement of the acoustic emissions.

On the other hand, in the case of the pTOFs measured between slave nodes, e.g., node *q* and node *l*, in the slave node *q* after the detection of the *Master Request* it is possible to measure the following pTOF after receiving the *Ack. Node l*:

(2) $${\hat{t}}_{q-l}=\frac{\left\|{\text{p}}_{M}-{\text{p}}_{l}\right\|}{c}+\frac{\left\|{\text{p}}_{l}-{\text{p}}_{q}\right\|}{c}-\frac{\left\|{\text{p}}_{M}-{\text{p}}_{q}\right\|}{c}+tp_{l}+T_{\text{CODE}}$$

where *t̂_q_*_−_*_l_* is the pTOF measured in the slave node *q* regarding the slave node *l*.

In (1), the computed pTOF depends on the distance between the *Master* and every slave node involved (‖**p***_M_*-**p***_l_*‖ and ‖**p***_M_*-**p***_q_*‖), and on the distance between the slave nodes (‖**p***_l_*-**p***_q_*‖). In this case, the temporary relation obtained in (2) only depends on the time delays of a slave node (*tp_l_*).

## 3. Positioning Algorithm

According to (1) and (2), to determine the node positions, it is necessary to solve a non-linear equation system. The parameters to be estimated are described in (3), i.e., the coordinates of every node as well as the time delays associated to the signal processing algorithms in the emission and reception stages:

(3) $$\begin{array}{l} \rho =\left[\text{p}\;\text{tp}\right]=\left[{\text{p}}_{M}\;{\text{p}}_{2}\;\cdots \;{\text{p}}_{q}\;\cdots \;{\text{p}}_{Q}\;tp_{M}\;\cdots tp_{Q}\right]\\ \rho =[x_{1}\;y_{1}\;z_{1}\;x_{2}\;y_{2}\;z_{2}\;\cdots x_{Q}\;y_{Q}\;z_{Q}\;\cdots \;tp_{M}\;tp_{2}\;\cdots \;tp_{Q}] \end{array}$$

where **ρ** is the parameter vector to be estimated; **p**_q_ are the coordinate positions (*x_q_,y_q_,z_q_*) for the node *q* and the vector **tp** represents the delay times associated to every node in the system, as described before.

**Figure 4.**
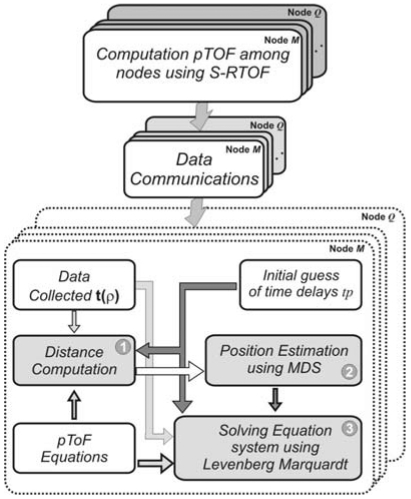
Positioning Algorithm Scheme.

The diagram of the algorithms used to compute the relative positions among nodes is shown in Figure 4. After applying the S-RTOF ranging method and assuming that the nodes have communicated the pTOFs computed by each one of them, the position can be obtained. The MDS algorithm provides a first approximation using the distances calculated with the pTOFs. Then, the results can be improved by using the Levenberg-Marquardt Algorithm (LMA), considering the MDS positions and the characterization of the time delays, performed by auto-calibration [19] or by off-line characterization, as initial point for LMA.

### 3.1. MDS Position Estimation

The MDS algorithm allows a geometric configuration of the object positions to be achieved in the smallest number of dimensions, by means of the Law of Cosines and linear algebra, when only the inter-node distances are known [6]. Before starting the MDS position estimation, the pTOFs measured in the different nodes are distributed among all of them. In this way, the data collected by every node can be described by (4):

(4) $$\text{t}(\rho )={\hat{t}}_{M-2}\cdots {\hat{t}}_{M-q}\cdots {\hat{t}}_{M-Q}\cdots {\hat{t}}_{2-3}\cdots {\hat{t}}_{q-l}\cdots {\hat{t}}_{(Q-1)-Q}$$

where *t̂_M_*_−_*_q_* are the pTOFs computed in the *Master* node for every slave node q; and *t̂_q_*_−_*_l_* is the pTOF computed between two slave nodes *q* and *l*, with *q,l* ∈ {2,⋯,*Q*} and *q* ≠*l*. The time delays *tp* are roughly determined at every node and they are considered time-invariants in the distance computation.

#### Computation of distances between Master and every slave node

Assuming that the moving velocities of the nodes are much lower than the propagation speed of the acoustic waves, it is possible to compute the distance between the *Master* and every slave node by the temporary relation described in (5), as follows:

(5) $$d_{M-q}=\left\|{\text{p}}_{M}-{\text{p}}_{q}\right\|=\frac{\left[{\hat{t}}_{M-q}-\left(tp_{M}+tp_{q}+T_{\text{CODE}}\right)\right]}{2}\cdot c$$

where *tp_M_* and *tp_q_* are the signal delay times in the emission and reception stage of the *Master* and the slave nodes respectively (*tp_M_* =*t_Spk M_* +*t_Mic M_* and *tpq* =*t_Spk q_* +*t_Mic q_*).

#### Computation of distances between every pair of slave nodes

On the other hand, with the data collected by the slave nodes, it is possible to compute their distances by (6) and the reciprocal pTOFs measured. Again, assuming that the node velocity is much lower than the propagation speed of acoustic waves, this distance is:

(6) $$d_{q-l}=\left\|{\text{p}}_{l}-{\text{p}}_{q}\right\|=\frac{c}{2}\cdot [{\hat{t}}_{q-l}+{\hat{t}}_{l-q}-tp_{l}-tp_{q}-2\cdot T_{\text{CODE}}]$$

where all the parameters have been previously defined.

If an additive Gaussian noise is considered in the measurements of pTOFs, in some particular cases, the slave nodes—see (2)—could detect a negative value of *t̂_q_*_−_*_l_*; in such a case the involved pTOF will be set to zero, which is the nearest possible value.

#### Computation of node positions

With all the computed distances, a matrix **D** can be arranged, as follows:

(7) $$\text{D}=\left(\begin{array}{cccc} d_{MM} & \ldots & d_{M(Q-1)} & d_{MQ}\\ d_{2M} & \cdots & d_{2(Q-1)} & d_{2Q}\\ \vdots & \ddots & \vdots & \vdots \\ d_{QM} & \cdots & d_{Q(Q-1)} & d_{QQ} \end{array}\right)$$

In order to obtain the node positions by MDS [6], it is necessary to build a matrix **B** called dot-product. This matrix considers the distances among objects from a reference point, being the most suitable the centroid (midpoint) of the figure formed by the objects in a two or three dimensional system (2D or 3D). Every element of **B** can be obtained as follows:

(8) $$b_{ij}=-\frac{1}{2}\left[d_{ij}^{2}-\frac{1}{Q}\sum_{q=1}^{Q}d_{iq}^{2}-\frac{1}{Q}\sum_{l=1}^{Q}d_{lj}^{2}+\frac{1}{Q^{2}}\sum_{m=1}^{Q}\sum_{n=1}^{Q}d_{mn}^{2}\right]$$

where *d_ij_,d_iq_,d_jl_,d_mn_* describe the distances among nodes with *i,j,l,m,n,q* ∈ {1,2,⋯,*Q*}.

Also **B** can be obtained from the position matrix **p,** previously defined in **(3),** but now considering the position matrix referred to the centroid as p*. In this way,**B** is obtained as follows:

(9) $$\text{B}=\text{p}*\cdot {\left(\text{p}*\right)}^{\text{T}}$$

It is possible to obtain the estimation of the node positions by the factorization of **B** using singular value decomposition (SVD), as follows:

(10) $$\begin{array}{l} \text{B}=\text{U}\cdot \text{S}\cdot {\text{U}}^{T}=\text{U}\cdot {\text{S}}^{1/2}\cdot {\text{S}}^{1/2}\cdot {\text{U}}^{T}=\left(\text{U}\cdot {\text{S}}^{1/2}\right)\cdot {\left(\text{U}\cdot {\text{S}}^{1/2}\right)}^{T}\\ \text{p}*=\left(\text{U}\cdot {\text{S}}^{1/2}\right) \end{array}$$

where **U** is the eigenvector matrix and **S** is the eigenvalue matrix. By selecting the first two or three columns of p* (with the largest eigenvalues), according to the dimensions (2D or 3D respectively) of the reference system, it is possible to obtain the estimation of the node positions. Finally, in order to map the estimated positions to the reference system of every node, since the obtained position results are referred to the midpoint of the objects, it is necessary to consider a rotation and a translation with matrixes that can be easily calculated.

### 3.2. Refining Results

The MDS provides a suitable estimation of the node distribution and positions by converting a whole matrix of distance measurements into a topology with two or three dimensions. Nevertheless, the exact object position cannot be achieved, since there are different error sources in the detection of acoustic signals (environmental effects such as room reverberation, acoustic transducer bandwidth, accuracy in hardware clock signals, etc). These errors corrupt the matrix **D** and imply inaccuracy in the position estimation. Assuming that the S-RTOF measurements are independently perturbed by a Gaussian noise with zero average and a variance *σ*^2^, the uncertainty of the observation vector **T** can be modeled as follows:

(11) T=t(ρ)+η

where **t(ρ)** is the vector of the true pTOF (4), which are a function of the parameters to be estimated **ρ;** and **η** is a noise vector where every element is associated to an observation from S-RTOF. According to this noise consideration, the density of probability function of vector **ρ** can be defined as:

(12) $$fd\;p_{\left(\text{T},\text{t}(\rho )\right)}={\left(2\cdot \pi \right)}^{\overset{-No}{\;}/\underset{2}{\;}}\cdot {\left|\sum \right|}^{\overset{-1}{\;}/\underset{2}{\;}}\exp-\overset{1}{\;}/\underset{2}{\;}\cdot {\left[\text{T}-\text{t}(\rho )\right]}^{\text{T}}\sum^{-1}[\text{T}-\text{t}(\rho )]$$

where **Σ** is the covariance matrix of noise vector **η;** and *No* is the total number of observations. The maximum likelihood (ML) estimator of parameters **ρ** is the one that maximizes the probability relation (12) [8], and it can be defined as:

(13) $$\begin{array}{l} \hat{\rho }=\arg_{\rho }\max F(\rho ,\text{T})\\ F(\rho ,\text{T})=-\overset{1}{\;}/\underset{2}{\;}\cdot {\left[\text{T}-\text{t}\left(\rho \right)\right]}^{T}\sum^{-1}\left[\text{T}-\text{t}\left(\rho \right)\right] \end{array}$$

In (13), the ML estimator depends on the observation vector **T**, as well as on the features or shape of the covariance matrix with dimensions *No*x *No*. An special case is when the measurement observations are independently perturbed, which means that **Σ** is diagonal, and the pTOF for the *Master* and the slave nodes have variances $\sigma_{M-q}^{2}$ and $\sigma_{q-l}^{2}$, respectively. This special case is usually used as an approximation of **Σ**; anyway a more detailed study about the nature of pTOF measurements should be performed in order to obtain the exact covariance matrix. According to this, if **Σ** is diagonal, the maximum likelihood estimator of parameters **ρ** has been obtained as:

(14) $$\sum_{q=2}^{Q}\frac{{\hat{t}}_{M-q}^{\text{Estimated}}-{\hat{t}}_{M-q}^{\text{Measured}}}{\sigma_{M-q}^{2}}+\sum_{q=2}^{Q-1}\sum_{l=q+1}^{Q}\frac{{\hat{t}}_{q-l}^{\text{Estimated}}-{\hat{t}}_{q-l}^{\text{Measured}}}{\sigma_{q-l}^{2}}=0$$

The first term in (14) represents the difference between the *Master* observations and its estimation, whereas the second term describes the difference between the pTOF observation and the estimation carried out between two slave nodes. According to this fact, the maximum likelihood estimator minimizes the difference between the measured times and the non-linear equations described in (1) and (2).

A suitable method to solve this kind of problems is the Levenberg-Marquardt Algorithm, which allows to find a local minimum by using a particular starting point. The results obtained with the MDS algorithm are a suitable starting point to estimate the coordinates by means of the LMA.

According to (14), the number of unknown coordinates as well as the time delays associated to every node should be considered. Nevertheless the estimation of all the time delays increases the complexity of the equation system to be solved so the time delays have been considered equal in all the nodes; under this assumption, the number of parameters to be estimated is:

(15) $$P=D\cdot Q+1-\frac{D\cdot (D+1)}{2}$$

where *D* represents the dimension of the coordinate system; and *Q* is the maximum number of nodes.

In order to solve the equation system, the number of observations carried out in the system should be higher than *P*. Since the number of linearly independent observations carried out by the proposed ranging mechanism is *Q*·(*Q* − 1) / 2, the minimum number of nodes required to determine the positions is:

(16) $$\frac{Q\cdot (Q-1)}{2}\geq D\cdot Q+1-\frac{D\cdot (D+1)}{2}$$

According to (16), if the positions are computed in 2D, the minimum number of required nodes is *Q* = 4; and in 3D it is necessary to have at least *Q* = 6 nodes in the system.

## 4. Simulations Results and Performance Analysis

In order to compare the accuracy achieved with the architecture previously described and the used positioning algorithms, some simulations have been carried out. In this analysis, different types of errors have been modeled.

### 4.1. Node Position Estimation Considering a Gaussian Error in the pTOF Measurements

**Figure 5.**
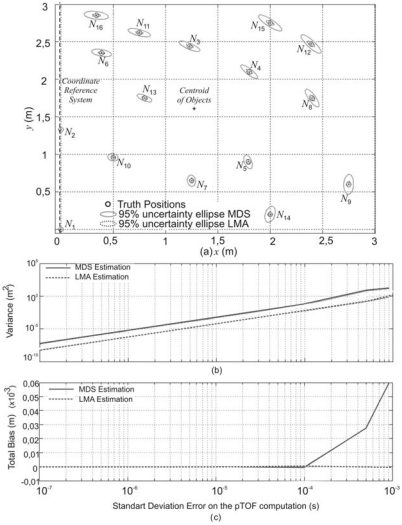
Position estimation for a distribution of nodes with a Gaussian noise in the pTOF measurements. (a) 95% uncertainty ellipses considering an error with standard deviation Σ = 100μs. (b) Total variance in the position estimation. (c) Total bias in the position estimation.

A first analysis has been performed by considering a node topology in 2*D* as shown in Figure 5, where the *Master* node condition is assigned to the object *q* = 1 with coordinates (0,0), whereas the node *q* = 2 forms a line with coordinates (0,*y*_2_), providing the coordinate reference system (see Figure 5a). In this case, every node has a pTOF vector **T** with all the temporary relations collected by the nodes of the system, which are distributed using wireless communication modules. Since disturbances such as ambient and electronic noise have Gaussian nature, it has been considered that every element of **T** is corrupted with an additive Gaussian noise with zero average and variance *σ*^2^. Furthermore, the time delays have been considered time-invariant and determined by an off-line characterization, with values *t_Mic_* = 50*μ*s and *t_Spk_* = 50*μ*s. The LMA has been carried out with the *lqsnonlin* function of the Matlab® optimization toolbox. One thousand tests have been done for every considered variance.

Figure 5a shows the 95% uncertainty ellipses (2*σ*) obtained around the estimated coordinate when the pTOFs have a Gaussian noise with a standard deviation *σ* = 100*μ*s. Using LMA for optimization it is possible to achieve a closer solution for the coordinate estimation, as shown with dotted lines in Figure 5a. Also, a characterization of the position algorithms through the total bias and total variance in the coordinate estimation has been carried out. In this case, different standard deviations in the measurement errors have been considered. As shown in Figure 5b, the results obtained by LMA have a smaller variance in the coordinate estimation for all the nodes. On the other hand, both estimators are unbiased, even in those cases where the considered standard deviation is *σ* = 100*μ*s (see Figure 5c), what implies an error in the distance measurements around 3 cm.

**Figure 6.**
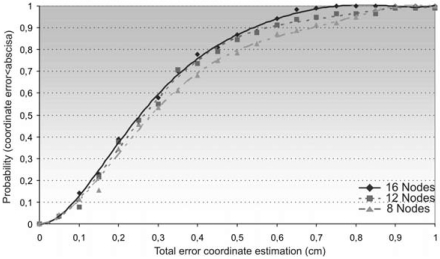
Cumulative distribution function in the position estimation of node *N_4_* depending on the total number of nodes (8, 12 and 16), and for a Gaussian error in the pTOF measurements of Σ = 10 μs.

If the positioning algorithms for *N*_4_ are computed changing the number of nodes in the topology described in Figure 6, i.e., considering 8, 12 and 16 nodes in every test, it can be observed that if the number of nodes is increased, the variance in the position estimation decreases. In order to verify this feature, the cumulative distribution function (CDF) of the positioning error has been obtained for the coordinate estimation of common node *N*_4_. The CDF is plotted in Figure 6 for different numbers of nodes, considering an error *σ* = 10μs in the pTOF measurements. In this case, if more node information is added, a higher increment of the cumulative effect is obtained, which means that a better precision can be achieved.

### 4.2. Node Position Estimation Considering Errors in the Signal Processing Delay Characterization

In the previous tests the signal processing delays have been considered time-invariant. Nevertheless, the true value of *tp* may be different from those used for LMA initialisation. Furthermore, they can be different from one node to another. These differences can come from the diverse execution times of signal processing algorithms at every node, since clock signals are not ideal and can have not exactly the same rates. Also, the differences in the response of the transducers may modify *tp* for every node.

**Figure 7.**
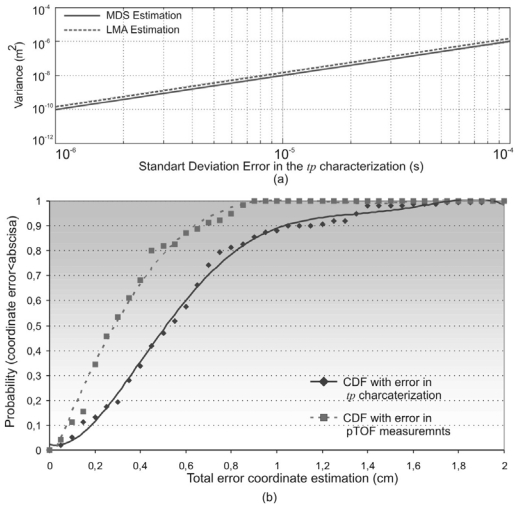
Position estimation with errors in *tp* characterization. (a) Total variance in the coordinate estimation of eight nodes. (b) CDF in the position estimation of node *N_4_*.

In order to verify the behaviour of the positioning process with these considerations, the algorithms have been tested with the topology described in Figure 5 and a Gaussian error in the signal processing delay characterization with a standard deviation from *σ_tp_* = 1*μ*s to *σ_tp_* = 100*μ*s (see Figure 7a).

In this case, it is observed that the MDS allows a higher precision than the LMA (see Figure 7a). On the other hand, in Figure 7b, the CDF for the position estimation of node *N*_4_ is depicted with a continuous line, only for errors in the determination of *tp*. Comparing it to the CDF obtained only for errors in the pTOF computation (dotted line in Figure 7b), it is observed that LMA is less sensitive to errors in the pTOF measurements than to errors in the *tp* characterization. This implies that the used positioning algorithm is less sensitive to errors in the node coordinates than to errors in the time delays.

### 4.3. Node Position Estimation Varying the Topology of Nodes

**Figure 8.**
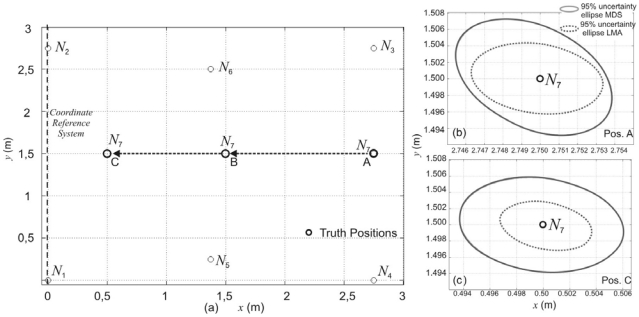
Position estimation when changing the topology of nodes.

In the previous analysis it is assumed that the node topology is fixed. In the topology described in Figure 8 node *N_7_* moves from point A, to B and C. At every position, the algorithms have been run one thousand times. The pTOFs have a Gaussian noise with a standard deviation *σ* = 10*μ*s.Figure 8b and Figure 8c show a good accuracy in the position estimation of the node *N_7_*. In this case, the orientation of the 95% uncertainty ellipse varies but the precision in the coordinate estimation does not depend on the node position referred to the reference system.

### 4.4. Effect of Non-Gaussian Errors in Node Position Estimation

The use of acoustic signals as ranging mechanism requires that the signal propagates along the line-of-sight (LOS) path between nodes. If LOS is blocked by obstacles, becoming a non-line-of-sight (NLOS) link, the signal may reach the receiver due to reflections in the environment. In this case the length of the reflected path is longer than the line-of-sight, reason why the measurements with NLOS errors can considerably bias the real pTOF value and provide inaccurate location estimation. These kinds of errors are non-Gaussian and they are more difficult to solve since they strongly depend on unknown environment conditions.

**Figure 9.**
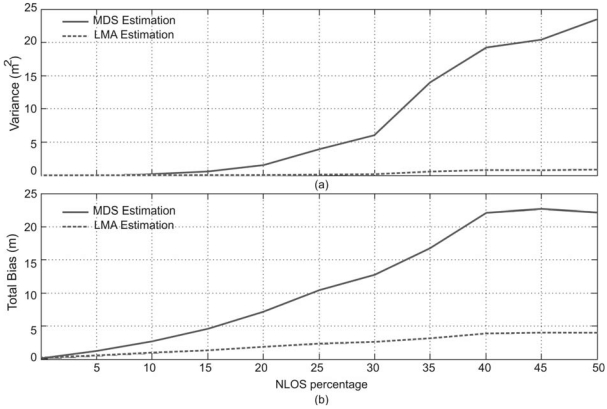
Errors in the position estimation considering NLOS effect between nodes *N*_1_ and *N*_14_ (see the node distribution in Figure 5). (a) Total variance in the coordinate estimation. (b) Total bias in the coordinate estimation.

In order to verify the performance of the proposed algorithms with NLOS effects, the topology of Figure 5 has been tested. In the first test performed, the distance measurements between nodes *N*_1_ and *N*_14_ have a large observation error, whereas other nodes have clear LOS path. In practice, this situation can happen when the LOS path between the considered nodes is blocked by walls or other signal-scattering object. The NLOS has been modelled as a positive bias error with an uniformly random distribution, where the maximum value is a percentage of the real pTOF measured in the interval [0%, 50%]. The total bias and total variance in the position estimation have been obtained for the different NLOS error considered as is depicted in Figure 9. In this case, the LMA estimation of node positions allows to obtain a suitable precision and a low bias in the global node position estimation, when the NLOS provides errors up to 35% over the real pTOF between the nodes *N*_3_ and *N*_6_. Nevertheless, when stronger NLOS effects appear between the considered nodes, the global node position estimation has a reduced performance with both algorithms.

**Figure 10.**
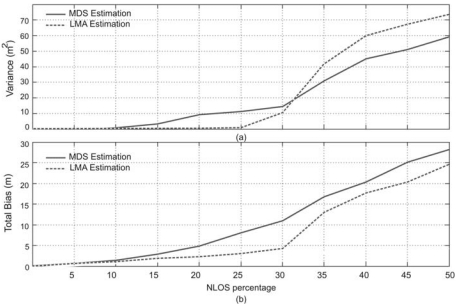
Errors in the position estimation considering NLOS effect between nodes *N*_1_,*N*_2_,*N*_6_,*N*_13_ and *N*_3_ (according to the distribution shown in Figure 5) (a) Total variance in the coordinate estimation. (b) Total bias in the coordinate estimation.

The second test considers an NLOS effect by means of a solid object closer to *N*_3_ (see the node distribution in Figure 5), which blocks the path from the nodes *N*_1_,*N*_2_,*N*_6_,*N*_13_ to *N*_3_. In this case, the total bias and total variance in the position estimation for different NLOS errors is depicted in Figure 10. It can be observed that the LMA estimation of node positions allows to achieve a suitable precision and a low bias in the global node position estimation, when the NLOS provides errors up to 25% over the real pTOF between the considered nodes.

This example shows that, due to the cooperative nature of the architecture, the NLOS effect in a set of pTOF measurement from different nodes implies errors in all the node positions. Nevertheless, the features of the ranging mechanism provide a set of redundant observation data, which, together with high-level algorithms such as geometry consistency or statistical analysis, can improve the estimation performance by identifying and rejecting those measurements with low accuracy.

## 5. Conclusions

The performance of a relative positioning algorithm based on the Multi-Dimensional Scaling (MDS) technique and the Levenberg-Marquardt algorithm (LMA) has been analyzed. In this case, the spatial relations among objects are determined by an acoustic sensor network where only acoustic emissions are used, providing a low-complex ranging mechanism. The MDS algorithm allows a geometric configuration of the nodes to be obtained with the minimum number of dimensions. Nevertheless, there are different error sources in the detection of acoustic signals that perturb the distance computation by inserting an error in the position estimation. In order to improve the achieved results, an optimization by LMA has been applied. In this way, the temporary relations among acoustic emissions carried out by the nodes have been used for the minimization. The characterization of the positioning procedure has been performed by Monte-Carlo simulations. The obtained results show that the LMA algorithm allows the precision in the MDS results to be improved, considering different node topologies and errors with Gaussian distribution in the determination of temporary relations. In this case, a fine-grained localization is achieved, even considering different error magnitude in the ranging mechanism (from *σ* = 1*μs* to *σ* = 100*μs*). The precision in the position estimation varies from 2 cm to 5 cm in the case of the MDS algorithm (when all the nodes are inside a square of 3 × 3 m). Furthermore, a better precision has been obtained by the LMA optimization: from 0.5 cm to 2 cm. On the other hand, the positioning algorithm is sensitive to errors in the signal delays *tp*, providing a high coordinate estimation error. Therefore it is necessary to accurately determine them by an online characterization and by considering them time-variant. Finally, non-Gaussian errors (i.e., NLOS effects) have been considered since acoustic signals are sensitive to solid objects. The obtained results show that the system is robust to a bias of up to 25% over the pTOF measured with LOS.
